# Silence, shame and abuse in health care: theoretical development on basis of an intervention project among staff

**DOI:** 10.1186/s12909-016-0595-3

**Published:** 2016-02-27

**Authors:** Barbro Wijma, Anke Zbikowski, A. Jelmer Brüggemann

**Affiliations:** Gender and Medicine, Department of Clinical and Experimental Medicine, Faculty of Medicine and Health Sciences, Linköping University, 581 83 Linköping, Sweden; Department of Obstetrics and Gynaecology, Ryhov County Hospital, Jönköping, Sweden; Current address: Department of Thematic Studies - Technology and Social Change, Faculty of Arts and Sciences, Linköping University, 581 83 Linköping, Sweden

**Keywords:** Sweden, Abuse in health care, Forum play, Silence, Shame, Regret, Health care intervention, Professional education, Moral learning

## Abstract

As health care exists to alleviate patients’ suffering it is unacceptable that it inflicts unnecessary suffering on patients. We therefore have developed and evaluated a drama pedagogical model for staff interventions using Forum Play, focusing on staff’s experiences of failed encounters where they have perceived that the patient felt abused. In the current paper we present how our preliminary theoretical framework of intervening against abuse in health care developed and was revised during this intervention.

During and after the intervention, five important lessons were learned and incorporated in our present theoretical framework. First, a Forum Play intervention may break the silence culture that surrounds abuse in health care. Second, organizing staff training in groups was essential and transformed abuse from being an individual problem inflicting shame into a collective responsibility. Third, initial theoretical concepts “moral resources” and “the vicious violence triangle” proved valuable and became useful pedagogical tools during the intervention. Four, the intervention can be understood as having strengthened staff’s moral resources. Five, regret appeared to be an underexplored resource in medical training and clinical work.

The occurrence of abuse in health care is a complex phenomenon and the research area is in need of theoretical understanding. We hope this paper can inspire others to further develop theories and interventions in order to counteract abuse in health care.

## ᅟ

### What is abuse in health care and why study it?

Health care exists to help and alleviate patients’ suffering and should thus not be inflicting unnecessary suffering on patients, and yet this happens. The topic is not often spoken about and when it is discussed a wide range of terms are used, e.g. abuse in health care (AHC), patient dissatisfaction, medical errors, and suffering related to or caused by health care [1]. In this article we will focus on AHC, which we define as failed health care encounters in which patients feel abused and suffer [2], or when staff have reasons to assume that patients feel abused. We presume, in line with other studies, that most acts of AHC are forms of unintentional harm [2, 3]. AHC may include a large variety of incidents, from a comment that felt legitimate for staff to utter but which was perceived as humiliating by a patient to e.g. physical violence from staff to administer an urgent injection a patient verbally refuses. It may be a subtle issue for staff to recognize when a patient feels abused and probably those events often even pass unnoticed by staff [4], which is reinforced by patients’ silence as to what they experienced [5]. A large number of studies have identified the existence of AHC in several health care settings. Some patient groups seem to be at greater risk for abuse, including children, individuals with learning disabilities, older people, or patients with a background of other kinds of abuse [6–8]. However, our studies also confirmed the existence of AHC in general patient and population samples in the Nordic countries [9–11].

As AHC implies suffering and is unacceptable for a health care organization, after more than a decade of empirical studies among staff and patients our research group designed and evaluated a model for staff interventions. The model focused on staff’s experiences of encounters in which they perceived patients experiencing abuse, and was based on a theory about what contributes to the prevalence of AHC. Later we have also tested the model with staff in a different setting.

### Why this article?

The present article describes our present theoretical framework for the occurrence of and counteracting of AHC and how this framework emerged during our work with interventions against AHC. The basis of the present framework is four-fold: 1. the initial theoretical framework; 2. our earlier studies of prevalence of AHC, and of experiences of AHC among staff and patients; 3. our clinical experiences of handling AHC; and 4. the development and evaluations of interventions among staff. The character of this article is therefore theory development. We will illustrate our experiences and theoretical assumptions with cases and citations that appeared during the workshops or have been published in earlier articles from our research group.

### The initial theoretical framework

#### Abusive incidents occur in a context

Galtung’s “vicious violence triangle” uses the analogy of the three corners of a triangle, which all three have to be in place to form a triangle, to illustrate how one corner, depicting direct events of violence, cannot be understood without at the same time also analysing the two other corners; depicting structural and cultural violence [12]. He argues that direct events of violence, which is what usually is recognized, are legitimized and nurtured by structural violence (e.g. hierarchies) and cultural violence (e.g. ideologies), and that generally speaking there is a causal flow from cultural via structural to direct violence. Galtung’s reasoning is supported by knowledge from social psychology, where Zimbardo in a similar way underlines the importance of situations and systems for the expression of violent acts [13].

Transforming this reasoning to the current field means that if AHC is regarded as the direct event corner of the triangle, those direct events of AHC would not occur unless there was a climate and clinical setting indirectly enabling AHC to take place. Cultural norms have an impact on what constitutes AHC as “[v]iolence within health-care settings often reflects dynamics that are broadly prevalent in society” ([14], p. 1683). A clear example of this is a study by Jewkes, Abrahams and Mvo of patient abuse in South-African obstetric services, where the authors identified a class and racial struggle as an important reason for the abuse [15]. This struggle should be understood in the context of the legacy of South-African apartheid. When Galtung’s approach is applied to AHC, it emphasizes the joint responsibility of all employed staff including the management, in dealing with abuse.

With this theoretical basis we assumed that direct events of AHC are nurtured by cultural and structural aspects, and therefore all three should be parts of an intervention model.

#### AHC as erosion of individuals’ moral resources

To understand how the violence triangle operates on an individual level we turned to works in moral philosophy and found Glover’s thorough analysis of how “ordinary people” can perform inhumane deeds, portrayed in his book *Humanity* [16]. Though Glover’s analysis centres on major atrocities that took place during the 20^th^ century, the mechanisms he describes can be transferred to other contexts, e.g., health care settings [17]. In short, Glover finds that it is the erosion of people’s moral resources (respect, sympathy, and moral identity) that may lead them to perform inhumane deeds. When applied to health care these mechanisms would include e.g. the fragmentation of responsibility (common in hospital settings with many subspecialties), distancing to others (often technology-aided), fear (e.g. of negative reactions by others), the imposition of a belief-system (e.g. an overriding claim to reduce cost), or moral slide (a gradual change towards inhumane behaviour with a pace so slow that the change often passes unnoticed). Any of these mechanisms can override and distort health care staff’s moral resources. These resources should be cared for, protected and “cultivated” by the organization, as they constitute a power to resist moral erosion, and therefore may contribute to a lower risk for AHC.

With this theoretical basis we assumed that direct events of AHC could be attributed to health care staff’s eroded moral resources, and an intervention model against AHC should therefore be based on efforts to strengthen staff’s moral resources.

### Patient and staff experiences of AHC

#### Operationalization in surveys among patients and prevalence accordingly

In our survey studies we operationalized AHC by means of three questions in the NorVold Abuse Questionnaire (NorAQ), which was developed in the 1990s in order to study lifetime prevalence of physical, emotional, and sexual abuse, as well as AHC in the Nordic countries [18, 19]. The abuse items of NorAQ have been validated for Swedish men and women [20, 21].

Studies using NorAQ have shown high life-time prevalence of AHC; 19.7 % in a Swedish female gynaecology patient sample, and 8 % in a Swedish male out-patient sample [10, 11].

#### Patient experiences

In qualitative studies, both male and female patients emphasized a loss of their human value. Women seemed to turn these emotions inwards and felt powerless, ignored, and that they were treated with carelessness and non-empathy, which could be summarized in the core category “nullified” [22]. Men perceived a crisis in confidence in the health care system, and felt ignored and frustrated, and these emotions could be summarized in the core category “mentally pinioned” illustrating the men’s inability to act according to their own interests [23].

#### Staff experiences

In qualitative studies among staff at a Department of Obstetrics and Gynaecology (at which we later organized an intervention; see below), staff’s understanding and awareness of patients’ experiences of AHC were explored: staff defined AHC as an ethical failure, while at the same time they did not take on responsibility; the incidents were seen as “ethical lapses” [4]. Staff’s awareness of AHC was dependent on the situation and staff’s room for manoeuvre, rather than being something staff had or had not [24]. In contrast to patients, who gave vivid narratives with many concrete details, staff’s examples were few, vaguely formulated and seldom self-experienced.

The results from these studies made us conclude that it was an urgent task to develop an intervention model and evaluate its potential effects.

### Clinical experiences of handling AHC

From clinical experiences we knew that some major pedagogic problems emerge when involving staff in discussions on how to handle situations where patients have felt abused:

I. When staff is confronted with the fact that their patient experienced abuse, this is contrary to what they aimed for, which may create “*self-defence” reactions*, such as dismissing the problem in an aggressive way or defending the abusive action (“I didn’t do anything abnormal”. “We had done everything that possibly could have been done thus it was correctly dealt with” [4]). In a discussion with the patient who felt abused, such strategies from staff easily become destructive for the patient, who then may feel re-abused [25].
II. Situations in which staff felt they did something morally wrong are prone to being suppressed/”*forgotten”*, which may be another way of handling what happened. This often interferes with staff's chance to learn from mistakes; increasing the risk of repeated, abusive behaviour [26].
III. One reason for negative reactions among staff is that what happened is left to the *individual.* As AHC mostly is a non-topic, staff may presume that they are the only ones in the workplace having displayed such misconduct.
IV. *A focus on mistakes and how to eliminate bad behaviour* is seldom as productive as a focus on good behaviour and on possibilities to expand it, combined with positive reinforcement for every little step forward on that path [27].

According to our clinical experience an intervention against AHC therefore should include ways to handle oblivion and negative reactions to abusive events happening in the past, and at the same time look for ways to intervene by stimulating good efforts in group processes. Therefore, we searched for an intervention which could: 1. Transform AHC to a “hot topic” at the index clinic. 2. Reshape the problem from an individual one associated to negative reactions to one that could safely be displayed for others in a joint search for morally acceptable solutions. 3. Train groups of staff in handling AHC constructively.

### The development and evaluations of interventions among staff

#### The model

In order to tackle the theoretical assumptions and empirical data described above, we turned to Forum Play (FP), a Swedish modification of the Theatre of the Oppressed (TO), developed by Boal as a tool to fight social injustices and increase people’s ability to liberate themselves from oppression [28]. In the TO context the idea of change is central, and focus is especially on how to accomplish change on any level from a position of being oppressed and deprived of power in a system characterized by structural oppression. In FP, interactive improvised role play is combined with reflection and value-clarification [29, 30]. FP has previously been shown to be a valuable method for reflective learning in care settings [31]. In Table 1 below we summarize the procedures of the model we used.

**Table 1:** Procedure during a Forum Play workshop

• Information about FP and the workshop • All participants recollect episodes of AHC which they have heard of or been involved in • The episodes are narrated to the rest of the group • The group chooses 2–3 episodes to work with • Subgroups create short role plays, which are shown to the rest of the group to demonstrate the moral dilemma^1^ and who is facing it^2^ • Spectators are encouraged to intervene as soon as they have an idea about an alternative way of acting and then try that out in the role play • In these trials, body language is particularly in focus • Actors in the role play and then spectators give feedback on the effects of the new alternative • The procedure is repeated until the group feels that among all played alternatives was at least one they could have chosen themselves had they been in the illustrated moral dilemma^1^ • Discussion about what happened during the workshop	

^1^“Moral dilemma” in this context means that the person facing the dilemma saw no ethically acceptable way out of a situation in which she/he perceived that a patient was being maltreated

^2^In our intervention with staff, the focus was on replacing the person facing the moral dilemma; who usually was a bystander staff member

The processes taking place in FP can also be understood in terms of Cognitive Behavioural Therapy (CBT). Behaviour experiments constitute a very powerful therapeutic strategy and the way we used FP has many similarities with such strategies: when a behaviour of the client is dysfunctional, a new behaviour is searched for which could eliminate the negative consequences of the old behaviour. The new behaviour is then tested in an experiment. If the new behaviour is followed by the desired consequences, this demonstrates to the client the need to revise earlier presumptions [32]. In FP, new behaviour is tested in action and evaluated, followed by scrutinizing of the presumptions on which the old behaviour was based.

#### Empirical results from evaluations of the intervention project among staff

At a Department of Obstetrics and Gynaecology (OB/GYN clinic) in the south of Sweden, we performed 16 workshops with FP during a one-year period. A typical workshop lasted 3–4 hours and was run by a drama instructor. About half of all staff at the clinic, 56 % (76/136), participated in at least one workshop. Participation was voluntary but took place during work hours and was strongly recommended by the head of the clinic. The intervention study was approved by the regional ethical review board in Linköping, Sweden (reg. no. 194–06).

A series of both qualitative and quantitative studies that analysed the impact and meaning of the intervention project have been published. Except for the two pre-intervention studies [4, 24] which have been cited above, three studies took place post-intervention, which enabled us to identify changes in staff’s perceptions of AHC during the intervention period [33–35]. One of the post-intervention studies was performed by an external evaluator [35].

In post-intervention interviews, staff showed stronger empathy with patients when talking about AHC than before the intervention. They also gave fewer explanations, justifications and trivializations [33], and their standpoint towards AHC revealed a stronger responsibility and more moral imagination than in the pre-intervention interviews [34]. The findings of an external evaluator included examples of how staff had behaved differently and more “courageously” after the intervention, had felt more self-confident in finding ways to confront colleagues who abused a patient, and had an increased wish to receive feedback from colleagues about their own behaviour (Citation 1) [35].

Citation 1

**Table:** 

“If I feel, see or hear about something *(like abuse)* I have no problem acting on it. I can’t stand walking around with these things gnawing at me” (p. 58) “Before this project I did not have so many choices of how to act, which made me not act at all. Now I feel much more secure in acting” (p. 59)	

The qualitative post-intervention studies [33, 34] also confirmed that the silence surrounding AHC had been broken. Increased awareness and more daily conversations among staff made the existence of AHC a shared problem. At the clinic, talking about and acting against AHC “had become ‘the right thing to do’” ([33], p. 7), and staff no longer accepted that AHC passed unnoticed.

The two drama instructors who led the FP workshops have each published a report containing detailed reflections on the processes taking place during the workshops [36, 37]. On top of detailed descriptions of their theoretical stance and their drama methods, they both describe scenarios that were used, what happened in the room, and how staff was affected by what happened.

Finally a thesis has been published, evaluating the intervention as a whole, including a quantitative evaluation [38]. All staff at the clinic filled out questionnaires, evaluating their attitudes toward AHC and willingness to take action when abuse was heard of or witnessed. The times of measurement were before, during, and after the intervention (five months, 14 months and 25 months after the first workshop). The questionnaires contained questions about matters such as the perceived impact of AHC, the experience of FP, and the perceived impact of FP. Matched pre- and post-data was tested for differences on relevant items.

In the quantitative evaluation, staff who participated in FP reported an increased ability to act according to their moral beliefs [38].

#### The research team’s experience of carrying through the intervention

The intervention was a pilot project and was introduced as such at the index clinic which may have increased potential participants’ hesitance to take part in the workshops. This was further reinforced by the stance taken by the head of the clinic and the drama instructors: taking part had to be voluntary. In contrast to other projects at the clinic aiming to improve quality of care, the message of voluntary participation may have made the intervention project somewhat dubious. This was further underlined by the character of the workshops, where the participants may have feared that they would be forced to “play theatre”. For the individual staff member a choice was introduced and the alternative to participate became more of an individual challenge than if all staff were to take part.

At another three clinics, where we tried to introduce the project in order to perform a replica, similar reasons, but now on an organizational management level, may have contributed to inhibit the clinics’ participation even before we had met any staff members.

Staff generally reacted very positively on the transformation of AHC to a task which the whole staff group carried responsibility for, and sometimes this approach relieved an almost tangible tension in the group. During all workshops the initial round among the participants revealed that everybody had memories of when they had been bystanders or perpetrators of abusive incidents in which they had not acted according to their inner moral. Thus all participants soon realized that they shared such experiences with everybody else in the room. A feeling of belonging to a safe group emerged during the workshops, based on the sharing of having been in AHC situations and of the same values and ethical stances taken towards AHC. The participants also realized that together they were able to find many alternative ways of acting other than the abusive one, or not acting as a bystander, and that they could benefit from sharing these experiences and helping each other out.

This made the research team reflect on the issue of shame and guilt that seemed to have been handled as individual problems in the climate that had prevailed at the clinic so far.

The drama instructors gave instructions about how to use one’s body to reinforce the message one wanted to convey and these were regarded as extremely helpful by the participants who generally were astonished over which effects they could achieve in precarious situations merely by reflection on which gestures, body posture, body placing and voice they wanted to use and by acting accordingly. How to practice a desired body language seemed to the participants to be an underutilized source of strategies for resolving AHC situations.

Acting out the ideas that came up during the role plays was a similar issue which was regarded very instructive by the participants and much more so than just analyzing and discussing an AHC situation. By acting out participants learnt to register what they felt in their own bodies when acting, and the co-participants reflected back how different bodily actions influenced bodily reactions on their part. This pedagogy also emphasized the message “you need to act” in future real AHC situations.

It became evident that the theoretical concepts (the vicious triangle and moral resources) we had used as a framework were pedagogically well suited. Staff quickly adopted them and started to use them and they seemed to function well in the communications about the complicated processes we handled.

### Discussion of the results of the evaluations in relation to the initial theoretical framework

When evaluating our results as a whole we turn back to our original theories (Galtung and Glover) to revise our initial assumptions. First we draw on data and experiences from FP workshops and relate those findings to the original assumptions. In the Theory Development section we develop further the theoretical fundaments of our research on basis of what was learnt during the interventions.

#### Data and experiences during FP workshops in relation to the original assumptions

The direct events of AHC could be regarded as nurtured by the culture at the OB/GYN clinic and our intervention worked not only by giving staff alternative ways to handle direct events of AHC, but seemed also to have influenced the culture at the clinic regarding AHC.

When staff had the opportunity to work together in groups and find alternative ways of acting against AHC, they together created a climate during the study period in which AHC was recognized, much talked about and deemed unacceptable, i.e. influencing the prevailing culture. It may be presumed that if this change of cultural norms continues over time, staff would in the long run question rules and policies imposed on them which they feel are unacceptable, and thus not only culture but also structures may change. Seeking for alternative ways to act against direct events of violence may enable a group to reveal oppressive structures, thereby offering possibilities to tackle and change these structures [28].

What came true of these presumptions? There were indications that silencing of AHC was no longer accepted by staff and that individual responsibility for the occurrence of AHC had changed into being a problem staff as a group felt responsible for and which needed counteraction. These changes signal cultural changes at the clinic, at least temporarily.

Structures at the clinic seemed however to be resistant towards change, at least during the study period. Even if a work group was established with staff from all professions and with the task to work with questions of failing patient encounters including AHC, this group had no support from the clinic management, could not effectuate changes and died out without the management giving the show away.

The workshops revealed professional hierarchies often to be involved in staff’s inability to protect patients against abusive incidents by someone superior in the hierarchy (see Case 1). Even if post-intervention studies showed that staff had found new ways to confront colleagues (Case 2) [35] or report incidents of abuse to someone higher up in the hierarchy [33], we could not observe any changes in the hierarchical structures at the clinic, and could therefore not conclude that the intervention had influenced this structural aspect of AHC.

Case 1

**Table:** 

When the gynaecologists at the clinic had educational meetings, one of the most experienced midwives took over the telephone of the doctor on call and also met patients applying at the emergency ward for gynaecologic care. She was to tell the patients that the doctor was occupied at the moment and would come in a while. When some patients repeatedly asked to see the doctor, the midwife would contact the doctor and plead with him/her to come. If that did not happen, the midwife felt pressure from two sides and did not know how to act. Her morals told her to stand up for the patient’s rights and not accept the proposed lie, but her professional education told her to obey those higher up in the hierarchy. This was a case most participants in the workshops reported that they had experienced. The reporting of these situations at the workshops was accompanied with shame.	

Case 2

**Table:** 

“One of our patients was on the radiography ward for an acute X-ray during labour. She asked for permission to go to the toilet to pee, which a nurse refused. The patient then peed in her pants. When she came back to the delivery ward the assisting nurse there was upset and called the nurse at the other ward, who was encouraged to apologize to the patient. The nurse was ashamed and apologized and the patient felt rehabilitated. Staff at the radiography ward learnt something for future reference.” (p. 38)	

Staff’s moral resources were strengthened in the following way: when the shame connected to the incongruity of one’s own intentions and the negative outcome for the patient in suppressed/“forgotten” events of AHC was clearly displayed and handled constructively in the group, staff learnt that there were alternative ways of acting that felt morally acceptable in abusive situations – others than the only one they previously had employed.

In post-intervention interviews, staff showed stronger empathy with patients, more of moral imagination and they felt a stronger responsibility for taking action against AHC than before the intervention [33, 34]. In the report from the external evaluator there were many examples of staff’s more courageous acting in AHC situation [35]. And in the quantitative evaluation, staff who participated in FP reported an increased ability to act according to their moral beliefs [38].

Thus, we assumed that the intervention with FP seemed to initiate a process of counteracting erosion of staff’s moral resources e.g. by triggering emotions related to respect and sympathy and by stimulating the development of moral imagination [16].

A special case of increased ability to act was seen in instances where staff dealt with suppressed/“forgotten” events during workshops, which staff may have suppressed for a long time in order to avoid painful memories [24]. During one workshop, a midwife told a very personal story about an incident that had taken place more than 10 years ago, in which a patient had been hurt (Case 3). In the FP sessions, staff regularly recognized, when looking back at incidents of AHC in which they had been bystanders, that even if they at that moment had felt totally incapable of acting as they saw no way out, there had in fact been other and for the patient less destructive ways to act than the one chosen. We therefore regard their moral resources to have become strengthened by participating in the workshops.

Case 3

**Table:** 

During one of the workshops, a midwife suddenly remembered a situation she had been in ten years ago, and which she had “forgotten”. On the delivery ward, a patient in advanced labour had severe pain and was to be examined by the obstetrician to decide on the appropriate pain relief. During the examination the patient protested strongly about the unbearable pain caused by the way the examination was performed, and after a while told the doctor to stop. He answered brusquely that he had to finish the procedure to be able to offer her pain relief. When the patient continued to protest and screamed out that he had to stop, he got angry and harshly carried on with the exam. The midwife in charge of the patient did not interfere and at that moment she partly took the obstetrician’s part. After the exam the patient was crushed and unable to take an active part in the rest of the delivery. The midwife did not talk to the patient about what happened, neither during the delivery nor afterwards. For a few weeks she felt uneasy when remembering the situation, but afterwards it all faded away and she “forgot” what had happened.	

To summarize, we presume that the FP intervention could be regarded to strengthen staff’s moral resources and increase their ability to act according to their inner morality. The effects were clearly there on an individual level, and on a group level we could observe a change in attitude to AHC, implying that it had become acceptable and sometimes even the first choice to take a stand against it and act accordingly. The need to deal with AHC was now seen as “a stone in the shoe”, something that calls for action [33]. Changes in “structures” could not be documented.

### Theory development on basis of what was learnt during the interventions

#### What happens if nothing is done?

**Fig. 1:**
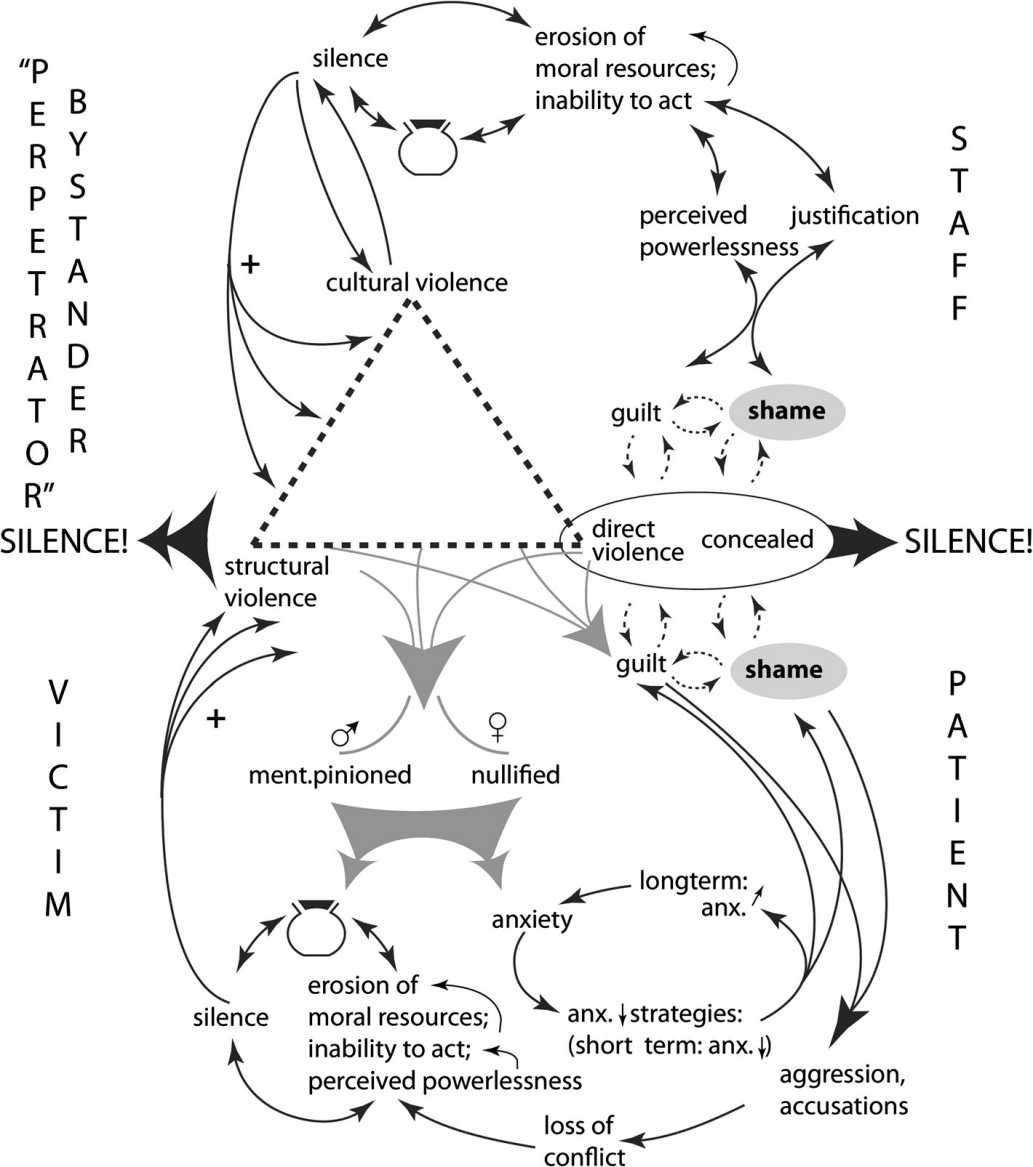
“What happens if nothing is done?” (Referring to the situation among staff and patients before intervention)

Figure 1 illustrates what happens in an AHC situation, using our initial theoretical concepts and combining them with the empirical findings from the intervention project. When AHC occurs, patients are victims and staff become “perpetrators”, even if they only take the role of silent bystanders. This fact is in itself provoking and may encourage the covering up of AHC incidents, like the midwife did in Case 3 (see above). In Fig. 1, Galtung’s vicious violent triangle is placed in the centre of the figure and it is suggested that events of abuse are often concealed by several mechanisms. Structures, e.g. hierarchies in health care, contribute; meaning that a staff member from a group that is inferior to the “perpetrator” should not try to intervene and stop the abusive situation, nor speak up about what happened (see Case 1). Culture in health care also helps in legitimizing abuse, most of all by denying its existence (Citation 2).

Citation 2

**Table:** 

“As we are there to help patients who suffer it is impossible that we can hurt and harm them.” (Citation from a young gynaecologist in training during a workshop)	

All the three corners of the triangle therefore fail to legitimise the experience of a patient, who is experiencing abuse and who is apt to feel nullified or mentally pinioned (lower part of Fig. 1) [22, 23]. Two common ways out of this frightful situation for the patient are silence and/or anxiety. If silence is chosen, the experience and the emotions associated with it are often suppressed; i.e. “put in a jar with a lid fastened on top” (see Fig. 1 lower part). Suppressing a situation that has been experienced as very abusive results in feelings of inferiority, powerlessness and an inability to act, and also the patient’s moral resources may be eroded – all contributing even more to silence [39]. When on the contrary, anxiety is a main strategy for the patient, it may be too strong to live through and the individual seeks anxiety-reducing strategies, e.g. drugs, self-mutilation, eating disorders, or avoids health care totally. Although such behaviours in the short run may reduce anxiety, using them will in the long run increase anxiety as well as guilt and shame over the behaviour [26]. Guilt and shame will then act as reinforcement on silence in a negative spiral [26]. A third strategy used by patients to handle guilt and shame is to become aggressive and accusing, which with untrained staff may lead to a destructive conflict, which patients are likely to lose [25, 40] (exemplified in Case 4).

When looking at the staff half of Fig. 1, their concealment of abusive episodes also creates guilt and shame, which reinforces their silence. These feelings may give rise to powerlessness or justification responses, which both however tend to erode staff’s moral resources and increase their inability to act according to their moral compass. As described for patients, concealed episodes of abuse that staff felt they did not handle correctly may be suppressed and may reinforce a culture of silence (see Case 3 and Case 4).

Case 4

**Table:** 

A is a 44-year-old man who as a child had been severely sexually abused and thereafter had felt abused in contacts with health care, when he had suffered from horrible flashbacks of the original traumatic situations while being rectally palpated. A had never talked to anybody about his early abusive experiences before his son’s birth. Shame, guilt, feelings of dirtiness, disgust with himself, inferiority and worthlessness had stopped him from digging the memories up, which was why these experiences had not been worked through. They were triggered with unexpected intensity in the delivery room when his wife had a vaginal examination to check the progress of labour. A then became totally overwhelmed and was lost in a chaotic world about which he could not communicate. He feared that if he told anybody about the strange fears he had, he would be considered psychotic, and thus be forced to leave the delivery ward. No one paid attention to his desperate state and he left the ward with the intention to commit suicide, as he could see no way out of the catastrophe he had landed in. A felt severely abused by the staff who did not recognize his dreadful situation but allowed him to leave the ward alone to go, as he then thought, to meet death. Afterwards his reaction was anger, intertwined with and originating from intense feelings of (unacknowledged) shame over what had happened in all those traumatic situations that now were afflicting him all the time, mentally and physically, day and night, and gave him no peace. His aggressive and confrontational behaviour was disrespectful and created defense reactions in health care staff, who answered disrespectfully. The staff’s reaction was negative as they felt they were accused of making medical mistakes, and they justified their way of acting, making A feel abused again. He interpreted their reactions as if they were saying: “We had the right to abuse you then and would do it again in a similar situation…”. A hopeless row of sessions all resulted in lack of conciliation. In this case, shame and silence played a major part in both the staff’s and A’s reactions, which is illustrated in Fig. 1. Even if the main person here is not the patient, similar reactions might also have been apparent in a delivering woman with a corresponding background.	

As Fig. 1 demonstrates, for staff as well as patients, the concealing of the abusive situation and its aftermath of silence may lead to erosion of moral resources, which increases the risk that patients and staff will run into a similar situation again and will once more be incapable of handling it in a constructive way. It also displays the central role of guilt (individual failure) and shame (a failure that becomes socially recognized) in nurturing silence and hence a “taboo” surrounding AHC. Thus, guilt and shame as consequences of abusive incidents are likely to increase a silence culture around AHC, which increases the risk of AHC occurring again in a vicious circle manner.

Figure 1 illustrates the individual perspective for staff as well as for patients, and the content of the figure can be illuminated by Case 4. The patient A in Case 4 illustrates that situation: several sessions, aiming at conciliation and alleviating the patient’s burden of unacknowledged shame [41], turned into new fights when A in an aggressive way expressed his desperation about the catastrophe that had struck him, which made the head of the delivery ward defend the actions taken by referring to medical facts (“justification” in Fig. 1) [25].

#### What happened after the intervention? “Breaking the silence and the effect thereof”

**Fig. 2:**
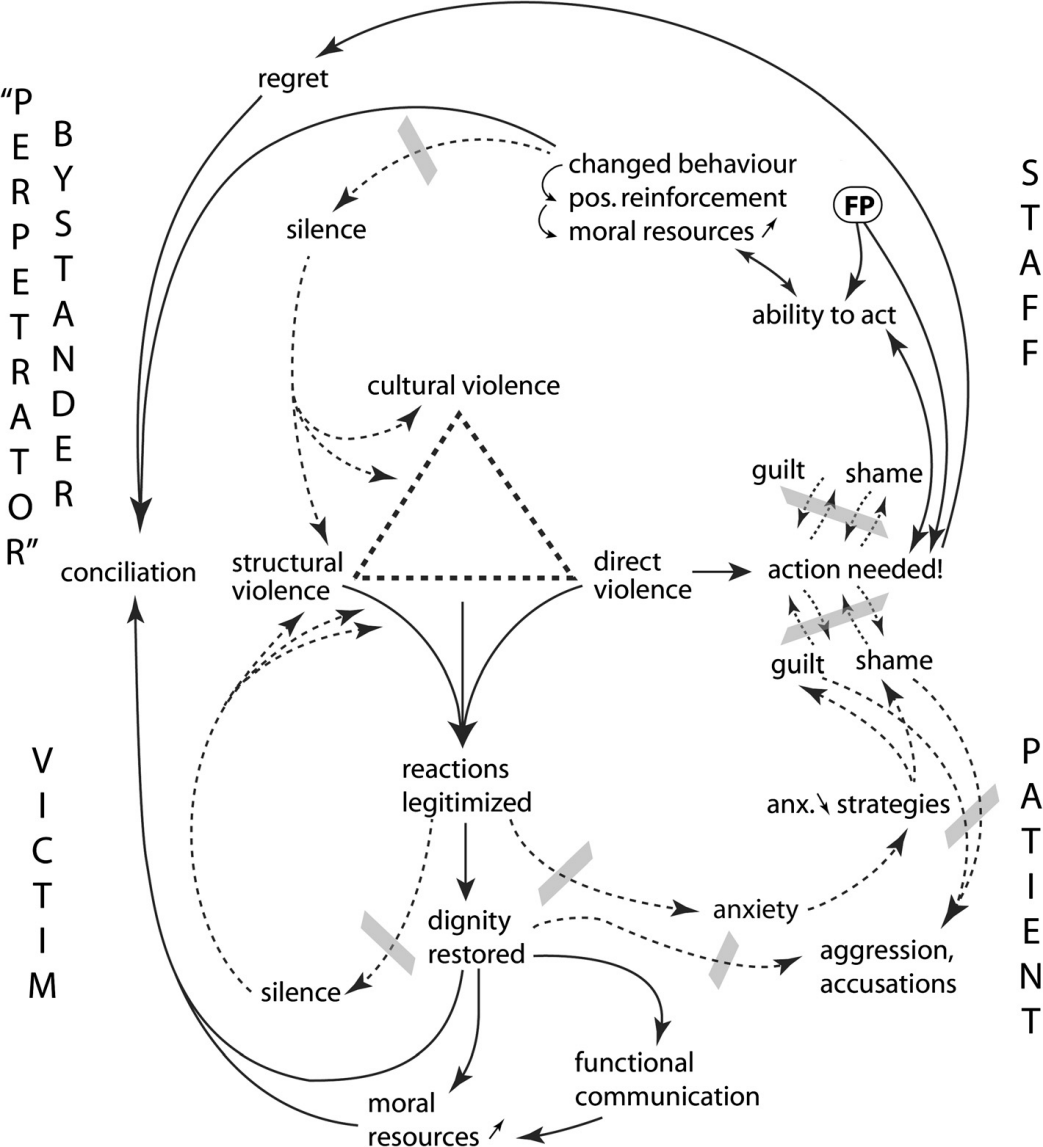
What happens when staff learns to act in abusive situations?

**Fig. 3:**
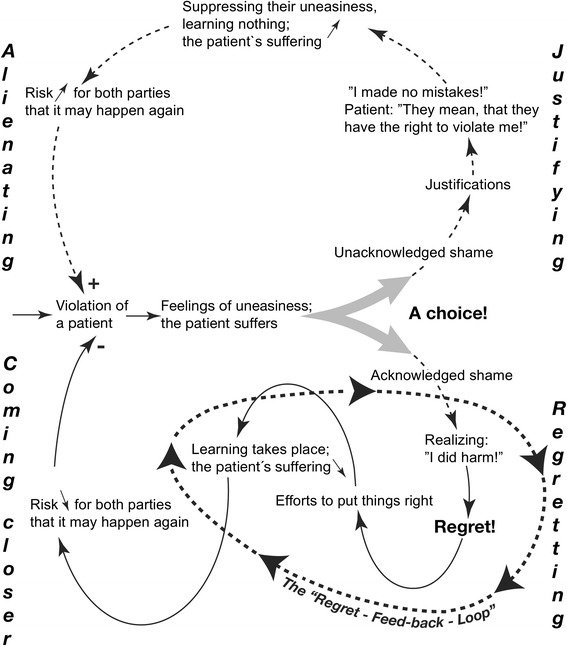
The “regret feedback loop”

Figure 2 is constructed in a similar way as Fig. 1. The new factor added is the training of staff with FP, thus adding group processes to the scenario. Staff had experienced during the workshops that even when the situation seemed hopeless and without any acceptable way out, many alternative ways of acting could still be created when the group worked together, and these ways could be tried out the next time they ran into similar dilemmas. Thus staff had learnt first of all that when an abusive situation occurs, action is needed; secondly that they knew of acceptable ways to intervene, and thirdly that they could support one another. Taking action reduced guilt and shame, which also increased their ability to express regret. It may be presumed that feeling competent in future awkward situations would strengthen their moral resources and increase their possibility to act. And for each time they did act, their new behaviour would be positively reinforced as the outcome of the situation most probably would turn into something positive for the patient and usually also for the “perpetrator”. When action was taken to end the abusive situation, the patient felt that she/he had been seen, her/his reactions legitimized, and her/his dignity restored. As the patient’s moral resources had thus increased, she/he was able to meet the abusive person and conciliation could occur (see also Fig. 3). These events could take place within seconds, and small acts on the staff’s side were able to create “wonders”, having empowering effects on both staff and patients [42].

Figure 2 shows how the impact of silence and shame which could be seen in Fig. 1 was reduced when staff as a group learnt to take action against AHC. One such case when a bystander took action is illustrated in case 2 above.

#### Shame

When shame operates without being acknowledged as such or properly named, it often creates behaviours that may seem inexplicable [41]. Scheff and Retzinger describe in their book *Emotions and violence: shame and rage in destructive conflicts* how human beings need secure bonding to important persons, which creates pride and is a prerequisite for functional communication and cooperation [41, p. 34-39, 65–69]. For patients the need of secure bonding to an important care giver is evident and the threat when bonding does not work is alienation, which easily is followed by shame. For staff, in a corresponding way, the bond to a patient is presupposed to function to everybody’s content. When confronted with their own wrong-doing, the bond not only to the patient is threatened but also to the colleagues, and the worst case scenario is being expelled from the community of staff, and shame will be a common reaction. For both parts, if shame is acknowledged as such, functional communication can occur and cooperation can take place concerning what happened and what could be done to find a way out. However, when shame is unacknowledged, it easily creates intense reactions, e.g. of anger or other types of disrespectful behaviour, which will disturb the communication and possibilities to cooperate. Disrespectful behaviour by one party is prone to create (unacknowledged) shame in the other, who may react in a disrespectful way, and there is a risk of vicious loop ([41], p. 65–69) (see Case 4). A full-blown conflict may occur between two persons who are caught by their shame reactions and have problems finding a constructive solution to the situation. When shame ***is*** acknowledged, on the other hand, respectful reactions may be the consequence and constructive cooperation more easily found [41].

However, there may be other reactions to unacknowledged shame than attacking others. Nathanson talks also about attacking oneself, avoidance, or withdrawal, as individual patterns of reactions to such shame [43]. An individual often uses the same strategy when repeatedly landing in such situations. Our patient A (Case 4) had used avoidance all his life, until the confrontation in the delivery room led to a breakdown of that strategy when he was drowned in his unacknowledged shame, and attacking others instead became his main strategy. This behaviour was totally inexplicable to the health care staff, who was unprepared to even try to see what factors were beneath his strong aggression. The final result of this conflict, which was based on unacknowledged shame by both parties, was – silence.

Shame and silence are so intertwined that it is difficult to separate them, as illustrated in Case 3.

#### The “regret feedback loop”

Figure 3 summarizes another theoretical direction that we explored based on FP workshops with staff. Many of the suppressed/“forgotten” episodes of AHC had already existed as an uncomfortable feeling, irritating now and then, but never allowed to be put in words, discussed or handled consciously. Staff repeatedly and frankly admitted that in their training they had never discussed how to handle feelings of regret. Regret could therefore be described as an underexplored moral resource in medical training and clinical work [37]. By regret here, we refer to the feeling of having done something (morally) wrong, and the will to learn from the incident in order to change future choices and behaviour.

In Fig. 3 we follow what happens when a staff member vaguely perceives that she/he has just violated a patient. The patient suffers from the incident and the staff member feels uneasy. This is when a choice appears: the staff member either acknowledges the uneasiness as shame and takes action (lower part of the figure) or does not acknowledge this and reacts with justifications (upper part).

When staff members acknowledge their shame, they realize that the patient had felt abused and regret their wrongdoing. This makes staff try to put things right, approach the patient and tell her or him that they are sorry for the inflicted harm. Most likely, the patient’s suffering will decrease, and staff will have learnt something, meaning that the risk has decreased that a similar abusive situation will occur again (see Case 2) [25, 44].

When unacknowledged, shame can make staff react with justifications of their behaviour, such as: “I made no (medical) mistakes!”, which are examples of the type of explanations given to legitimise AHC and which were seen in interviews from before the intervention [4]. This means that staff suppresses their uneasiness, learns nothing, and the patient’s suffering increases (see Case 4). The risk is then greater that staff will repeat similar behaviours than if they had taken the “regret feedback loop”; illustrated in the lower half of Fig. 3.

#### Limits of the intervention study

In this article, an empirical basis was used to display our theoretical development based on the intervention and to demonstrate what happened “on the floor” by means of cases and citations from the workshops and earlier publications. The developments are demonstrated in the figures. As they are theoretical interpretations based our research perspectives, there are pros and cons with this arrangement. For practical reasons and in order to learn as much as possible about the course and effects of the intervention, parts of the research team were present at many of the workshops. This led to a deepened understanding of the processes taking place, which we considered to be a greater advantage than the theoretical risk of changing the course of the workshops in particular directions.

In a study of FP training courses given to employees associated with the county council’s ethical committee, the research team was only very occasionally present during the course and the results were similar to those reported here [45]. This may support our assumption that potential bias created by our participation in workshops during the intervention probably had played a minor role for the outcome of the studies. In the study the core-category “developing response-ability” captured the essence of the participants’ experiences of the course [45]. These findings strengthen the developments presented in the current article, especially the observation that staff’s moral resources were strengthened during the study period and that they had developed the attitude that taking action against AHC was the right thing to do.

Even though we were able to document positive effects on an individual level and for some groups at the target clinic, we did not make great progress in our trials to influence policy documents or strategies on a higher level in the organization. Turning back to Galtung’s vicious violent triangle, we did not manage to influence the structural violence corner of the triangle, and probably only partly and temporarily the cultural violence corner.

## Conclusions

According to our present theoretical framework, what happened during and after the intervention can be described as: 1. the silence culture surrounding AHC was broken; 2. training staff in groups was essential and made it possible to transform AHC from an individual problem inflicting shame to a group responsibility; 3. “moral resources” and “the vicious violence triangle” became useful pedagogic tools in the intervention, 4. moral resources were strengthened during the study period, and 5. regret appeared to be an unexplored resource in medical training and clinical work.

As complex reactions take place when AHC occurs, in individuals as well as in groups and systems, there is an urgent need of theoretical development within the research field. We hope that our work and theoretical models inspire others to continue theoretical and pedagogical development in this under-researched area, in order to facilitate counteracting AHC.

### Ethics approval

The intervention study was approved by the regional ethical review board in Linköping, Sweden (reg. no. 194–06).

### Consent

Patient A has given his written consent for publication.

## Abbreviations

AHC: abuse in health care
FP: forum play
